# Nutritional Facts and Health/Nutrition Claims of Commercial Plant-Based Infant Foods: Where Do We Stand?

**DOI:** 10.3390/plants11192531

**Published:** 2022-09-27

**Authors:** Nicola Gasparre, Marina Mefleh, Fatma Boukid

**Affiliations:** 1 Department of Food and Human Nutritional Sciences, University of Manitoba, Winnipeg, MB R3T 2N2, Canada; 2 Department of Soil, Plant and Food Science (DISSPA), University of Bari Aldo Moro, I-70126 Bari, Italy; 3 ClonBio Group Ltd., 6 Fitzwilliam Pl, D02 XE61 Dublin, Ireland

**Keywords:** vegan, vegetarian, dairy, complementary feeding, cereals, food labelling

## Abstract

One of the current drivers of the infant food market is the rising demand for vegan products, and thus accurate knowledge of their nutritional composition is required to guide parents and health professionals. Thus, this study aimed to assess the nutritional composition of commercial plant-based infant foods, in addition to analyzing their health/nutrition claims. A selection of infant products launched in the global market (2017–2021) were classified into eight types and each type was divided into vegan and vegetarian products. Based on the ingredients list, cereals, seeds, pseudocereals and/or pulses were the most used ingredients in the retrieved products. The nutritional composition of six out eight types varied significantly among vegan and vegetarian products. When protein, calcium and iron contents differed significantly, vegan products had the highest protein content in all categories, compared to those that were vegetarian. When significant differences were found in sugar content, vegan products have lower amounts in all categories, compared to vegetarian products. Health and nutrition claims were found mostly used in vegetarian products. Strategies to reduce added sodium and sugar, and saturated fatty acids is required to ensure a healthy diet for infants. This study also implies the importance of a complete labelling of infants’ foods, especially vegan products to help parents making a reasonable choice.

## 1. Introduction

Proper infant nutrition is fundamental to a child’s continued health [1]. A correct diet in the first three years of life is important to promote healthy growth [2]. Several studies also reported that infant feeding practices can influence the onset risk of health issues, such as obesity, cardiovascular disease and type 2 diabetes [3,4]. Exclusive breastfeeding is recommended by the World Health Organization for about the 6 months or even longer (first 1–2 years) into an infant’s life [1]. If breastfeeding is not possible or supplements are needed, infant formula can be introduced to provide essential nutrients [5]. Beyond six months, complementary foods can be progressively introduced, and thus gradually replace breastfeeding and/or infant formula [2,6,7]. In 2019, the global infant food market size was valued at USD 67.3 billion, with a forecast of over USD 96 billion by 2027 [8]. In Western countries, the increasing number of working women is probably driving the demand growth for infant food. 

Commercial infant foods (referring to infant formula and complementary foods) are versatile, such as infant milk, infant juices, infant yoghurt, infant cereals, fruit purees, vegetable purees, and infant biscuits and snacks. They can be made from plant sources (e.g., grains, legumes, nuts, seeds, vegetables and fruit), and animal products (e.g., egg, dairy and meat). The wide diversity of plant-based ingredients, namely cereals, pulses, seeds and pseudocereals, provide manufacturers a large portfolio of ingredients to formulate products suiting different ages and needs. These ingredients have a rich composition of nutrients, such as protein, minerals and vitamins, and they are used in most countries in infant formulations [9]. Cereal and pseudocereals flours are generally used for infant cereal production due to their multiple health benefits and availability even in lower income countries [10]. Legumes are an alternative protein source to dairy or soy in complementary foods [11]. The general rule for introducing complementary foods is to provide sufficient energy, protein and micronutrients (Vitamin D, iron and zinc) to cover a child’s energy and nutrient needs at different ages [12]. Cow’s milk and dairy products are among the basic ingredients in infant foods owing to their high-value proteins, vitamins, minerals and fats [13]. However, the prevalence of vegan dietary habits and the number of vegan products on the market is surging worldwide and parents’ interest for this category is growing towards adopting vegan diets for their children [14,15]. Ethical, health and environmental considerations are fueling this shift [16,17]. Furthermore, there are medical reasons due to the association of cow’s milk and dairy products with allergies and intolerance to children. In a such case, children should follow a strict dairy-free diet. Prevalence data are lacking for vegan children. For instance, 1% of children aged 8–18 years are estimated to be vegan, as is 3.4% of the total American population [18]. 

Vegan products are foods that do not contain animal products, neither directly (e.g., meat, seafood, fish, chicken and lard), nor indirectly from living animals or their derived products (e.g., milk, cheese, butter, eggs or honey). Vegetarian products might contain indirect animal products but no direct products [19]. In general, vegetarian diets are presumed to be healthy, since they are a combination of plant-based and dairy foods [20]. Only meat and fish are excluded from the diet. However, the energy and nutrient requirements per kg of body weight of growing children is higher than adults, putting them at risk of nutrient deficiency, in particular omega 3, vitamin B12 and some minerals, of which bioavailability is compromised in cereals and legumes [21]. Well-planned vegan diets, balanced in nutrients and containing a variety of exclusively plant-based foods, can be appropriate for nutrition throughout all stages of life, including pregnancy, lactation, infancy and childhood [22,23]. However, as in the case of vegetarianism, guidelines for a healthy alternative diet for adults cannot be extrapolated to children. Vegan children were shown to be at risk of vitamin A, D, and B12; all types of fat; and essential amino acids deficits [24]. Even though few epidemiological studies on the safety and feasibility of vegan and vegetarians diets for infants are still lacking, it was reported that vegan children have a lower risk of developing obesity, type 1 diabetes and cardiovascular diseases [18,21,25,26,27]. However, if not correctly managed, vegan diet may lead to serious nutritional deficiencies, requiring specific nutritional supplementation to fulfill children (including infants and toddlers) needs at different stages [22]. Consequently, a deep understanding of the characteristics of a complete vegan diet is required by pediatricians and healthcare providers to guide parents to opt for an appropriate and complete diet for their children throughout all stages [14,28,29]. 

Currently, the vegan section of the infant food market is still a niche, with global launches representing around 1% of total plant-based launches [30]. In terms of nutritional evaluation, most studies focused on comparing the nutritional composition of milk to plant-based drinks dedicated to children and toddlers [31,32]. Scientific evidence showed that many of these milk alternatives do not necessarily address the nutritional requirements of infants and children [13,31,32]. Qualitative and quantitative nutritional compositions of infant products guide parents and medical providers in selecting the best options for children. To the authors’ knowledge, no focus was addressed towards vegan infant food categories to assess their suitability for feeding babies and toddlers. In the light of these considerations and facts, the aim of this study is to examine eight types of vegan infant foods sold in the global market, comparing them to their vegetarian counterparts. To do so, products launched from 2017 to 2021 were retrieved, and their nutritional facts (energy, protein, carbohydrates, fat, saturated fatty acids (SFA), sugar, fiber, iron, sodium and calcium), list of ingredients, nutrition and health claims were evaluated.

## 2. Results and Discussion

### 2.1. Descriptive Analysis

Product types and distribution: Based on Table 1, a total of 2195 infant foods were considered and were divided into eight types, i.e., baby formula (0–6 months), baby cereals, baby fruit products, desserts and yogurts, baby biscuits and rusks, baby snacks, baby formula (6–12 months), baby savory meals and dishes, and baby juices and drinks. The largest type is baby formula (0–6 months) with ≈26% share of total products followed by baby formula (6–12 months) and baby fruit products, desserts, and yogurts (≈18%) and baby cereals (≈16%). Baby juices and drinks has the lowest share (<1%). 

Among all the infant foods, only ≈8% was vegan (*n* = 185), in comparison to ≈92% of vegetarian products (*n* = 2010). This underlines that the vegan category is still a niche market. Considering each products type separately; the vegan products represent a limited part of the total considered products. As reported in Table S1, limited number of products did not contain dairy ingredients (whey protein, casein, milk derived products and cheese). Indeed, these ingredients are commonly used in infant formula to ensure adequate amounts of nutrients to infants [33,34].

Plant-based ingredients: Table S1 shows that cereals, pseudocereals, seeds and pulses are the main plant-based sources used in infant formulations. Cereals are used in most product types. The most used cereals are wheat, barley, rice and corn, as they are excellent sources of energy, proteins and micronutrients, which are important from the age of six months to cover the nutritional requirements of the infant [9,35]. Cereals ingredients are diverse, such as flours, fibers, extract, starches or proteins. Minor cereals (oat, barley millet and sorghum) are also used in infant formulations, owing to their nutritional value and their suitability for gluten-containing and gluten-free formulations. Oat is particularly gaining interest for its use as a milk alternative, owing to its rich composition in proteins [36]. 

Pseudocereals are naturally gluten-free and have a high protein content and quality [37]. Quinoa is used several categories, such as baby biscuits and rusks, baby snacks, desserts and meals. It is used mainly in two forms, namely flour and milk. Quinoa has been suggested in several follow-up formulas because it provides sufficient protein and other essential nutritional elements [38].

Lentil, pea and chickpea ingredients are frequently used in infant food formulations, such as baby snacks, biscuits and meals. Pulses are rich sources of nutrients, such as proteins, and they are naturally gluten-free. Therefore, they can suit specific formulations for infants with intolerances or allergies to gluten. In most cases, these pulses are blended with cereals to provide formulations with a balanced amino acid profile, since they contain relatively high quantities of the essential amino acid lysine, which are lacking in cereals. Yet cereals contain sulfur amino acids, such as methionine, which are deficient in pulses [39].

Oilseeds are used in different forms, namely oil, protein isolates and flours. Soy proteins are commonly used in infant formulations as a source of proteins with a complete composition of amino acids [40].

A wide range of vegetables and fruits are used in infant formulations, as they contribute to a balanced diet for infants [41]. Vegetables are prioritized for complementary feeding, also known as a “vegetables first” approach [42]. Green leafy vegetables, such as spinach, tend to be added at 9 months to an infant’s diet due to their composition being rich in nutrients, such as protein, folate and vitamin B. Introducing vegetables (e.g., pumpkin and potato) at early stages may promote acceptance (possibly limited by their bitter taste) through familiarization and facilitate intake throughout childhood [43]. In comparison to vegetables, fruits are generally more accepted due their sweet taste [44].

Vitamins and minerals are added to all formulations to provide infants with necessary micronutrients for their growth. 

### 2.2. Nutrition Facts of Different Commercial Infant Foods

Table 2 shows the nutrition facts of eight types of infant foods classified as vegan and vegetarian. Vegan baby formula (0–6 months) provided higher calories than those containing dairy ingredients. Fat and SFA contents were also found higher in vegan products, as well as sodium content. No significant differences were observed in carbohydrates and fiber content. The presence of dextrins and maltodextrins in this product category, especially in baby formula (0–12 months) assumes great relevance. These low-molecular-weight carbohydrates, obtained through the partial starch hydrolysis (acid or enzymatic) are mainly employed as thickening agent, to lower hygroscopicity and prevent crystallization in food processing. For this reason, they are key ingredients in infant food manufacturing because they help to achieve the adequate mouthfeel and viscosity [45]. Moreover, during the industrial process, heat treatment and hydrolysis with a-amylase increase their final content. In spite of that, their presence in the end products should be carefully assessed because their easier digestibility rises the glycemic index of the final products [46]. Sugar was found significantly higher in vegetarian products, compared to vegan products. The median value of protein was almost the double in vegan products, compared to vegetarian products.

Regarding baby formula (6–12 months), no significant differences were shown in energy, fat and carbohydrates. SFA and sodium contents were significantly higher in vegan products. Remarkably, the fiber content in baby formulas from 0 to 12 months was in the form of traces, which aligns with the recommendation that meals from children up 12 months should be fiber deprived [18]. Sodium content was found higher in vegan products for baby formula (0–12 months). Salt content was considerably high (170 mg/day) considering that the adequate intake of sodium per day for 0–6 months is 110 and 370 mg, respectively [47]. As for commercial infant cereals. The median value of SFA and protein of baby formula (0–12 months) was much higher in vegan products, compared to vegetarian products. High SFA might be due to the use of coconut oil and palm oil, which are relatively high in saturated fats [48], while the content of sugar was higher in vegetarian products.

For baby biscuits and rusks, no significant differences were observed in terms of energy, fat, SFA, carbohydrates, fibers and sugar among vegan and vegetarian products. Both types had high sugar and carbohydrates contents. This can be attributed to sources, such as cereals having high carbohydrate contents, starches and sugars used in the formulations (Table S1). This aligns with a recent study examining the list of ingredients showing that at least one sugar-contributing ingredient was used in infant formulation [49]. Consistently, sugar content was found high (up to 40 g/100 g) in infants cookies sold in Taiwan [50], commercial weaning foods sold in the UK [51], and products for infants and toddlers sold in the US [52]. This is in contradiction with dietary guidelines, recommending that parents should avoid added sugar intake until age two and limit sugar intake to 25 g per day for children aged two to 19 years [53]. Furthermore, the sodium amount in vegetarian and vegan products is high (median value of around 174 mg/100 g) considering that the adequate salt intake for children 7–12 months is 370 and 1–3 years is 800 mg per day [47]. These findings are consistent with previous studies reporting that infant cookies sold in Canada and USA have high sodium content (>130 mg) [52,54]. This suggests that parents should pay special attention to sodium contents when choosing, in particular, vegan cookies for their infants. Protein was slightly high in vegan products showing a high intra-variability that reflect difference in formulations of products sold in the market. It must be kept in consideration that beyond protein quantity, its quality is equally relevant, yet the composition of amino acids profile is not reported on products labels. From the list of the ingredients, we can understand that the exclusive use of cereals does not allow a complete profile of essential amino acids, and thus the combined used of cereals with pulses can enable the filling of the gaps and the obtaining of a complete essential amino acid profile in vegan products [31]. Blending nixtamalized maize flour (26.7%) with extruded chickpea flour (73.3%) allowed basic infant food with high protein quality and digestibility to be obtained [55]. 

Commercial infant cereals are among the first solid foods introduced to infants’ diet worldwide [35]. Results showed that energy, fat, sodium, carbohydrates and fiber contents of baby cereals did not show any significant difference among vegan and vegetarian products. SFA were found higher in vegan products. Sugar content was found to be much higher in vegetarian products. Previous studies evidenced that many infant cereals sold in Germany contain high levels of added sugar (up to 29 g/100 g) [56]. Interestingly, proteins were higher in vegan products, suggesting the food industry focus on improving the protein quantity of products exempt of dairy products. Vegetarian products had a high range of variability of proteins, probably due to the numerosity and heterogeneity of commercial products and the possibility to use a high range of proteins (plants and dairy based). 

Regarding, baby snacks, no difference in energy, carbohydrates, sugar, protein fat and SFA was observed. The median of sodium was higher in vegetarian products than vegan, despite having similar range of variability. Fiber was higher in vegan products despite the comparable range of variability with vegetarian products. Results from an in vitro study showed that infant gut microbiome structure and metabolic activity could be modulated by the fiber-derived sugar of some plant-based foods [57]. Nevertheless, attention to increasing the fiber content was recommended beyond 1 years of age to reach the adequacy of the diet [18].

The nutritional composition of baby fruit products, desserts and yogurts did not show significant differences among vegan and vegetarian products. These formulations were characterized by the absence of fat and SFA. Moreover, the calories derive chiefly from sugar, since it does not contain fats and only low amounts of proteins were found. Vegan baby juices and drinks provided higher calorie than vegetarian products. They also provided higher fat and SFA contents. This is probably due to the absence of diary ingredients that, in vegetarian products, can stabilize the system preventing phase separation. To overcome this problem, raw materials with higher fat content, such as flour from barley and millet, can be used (Table S1). Carbohydrate content was also found to be much higher in vegan products, which can be to the use of white sugar, maltodextrin and caramel, particularly at high amounts to improve the viscosity and enable a similar thickness to that of vegetarian products, containing dairy ingredients. Nevertheless, fruit juices with added sugar should be avoided due to association of excessive sugar rich foods with health concerns, such as obesity and being overweight [58]. Sodium, fiber, sugar and protein contents did not show significant differences.

The nutritional composition of baby savory dishes and meals did not show significant differences among vegan and vegetarian products. It is likely the use of cereals and vegetables in the formulation was much higher than dairy ingredients (reported in Table S1), and thus no significant changes were resulted from the addition of dairy ingredients. In this category, products were more balanced in terms of fat, SFA, sugar and sodium than the babies’ products.

### 2.3. Calcium and Iron Contents

Table 3 illustrates the calcium and iron content for different ages. Iron and calcium are two essential nutrients for infant health and development [59,60]. In the first case, iron is essential for the regular infant growth and for cognitive development, and a deficiency can lead to anemia and increased rates of mortality [61]. On the other hand, the formation of the bone structural matrix is strongly dependent on an optimal homeostasis of the calcium; furthermore, low calcium intakes have been associated with rickets in infants [60]. Such information is of high interest for parents to make better choices about the dietary adequacy of commercial products for their infants.

For calcium content, the recommended dietary allowance is 200 mg for 0–6 months, 260 mg for 7–12 months and 700 mg for 1–3 years. In all categories of vegetarian products, there is a noticeable high variation (0 to more than 500 mg per 100 g) in calcium content, except for baby desserts and yogurt and baby meal and dishes (max 67 mg/100 g). Vegan baby formula at stage 1 (0–6 months) had higher calcium content than vegetarian products containing dairy ingredients. No significant differences were observed in calcium content in baby formula stage 2 (6–12 months), yet the median value of vegan products was much higher than that of vegetarian products. Median values vegan infant cereals had higher calcium than that vegetarian and showed a high range of variability, which can be attributed the heterogenicity of commercial formulations (containing dairy ingredients, Table S1). Baby cereals showed similar content of calcium among vegan and vegetarian products. Although vegetarian products had high median value, no significant difference in calcium content among baby snacks. Notably, calcium amounts in vegan products are, in part, coming from biofortification to have comparable content to that of products containing dairy ingredients. Vegetables, such as spinach and broccoli, are also good source of calcium [62]. Baby fruit products, desserts and yogurts did not show a significant difference regardless of the formulation. For baby juices, no significant difference was shown regardless of the high median of calcium content in vegan products. The amount of calcium in baby savory meals and dishes did not vary, depending on the formulation and it was the lowest among all infant foods.

The iron content in baby formula at stage 1 and 2 was found higher in vegan products probably due to higher fortification amounts. In fact, iron requirements of these babies should be met through breast feeding and iron-fortified infant formula [63]. Iron content of baby biscuits and rusks did no vary significantly as a function of formulation. Nevertheless, the median value of vegan products was higher than vegetarian products. Iron is present in low amounts in milk and plant foods (whole grains, legumes, soy, green leafy vegetables, nuts and seeds) [2]. No significant difference was observed across baby cereals, since both types had similar ingredients, i.e., cereals and grains, which are probably present in higher amounts than dairy ingredients used in making vegetarian products. Furthermore, baby fruit products, desserts and yogurts, and baby juices and drinks were found the most in the vegan formulations. No significant differences were found among baby savory meals and dishes and baby snacks.

### 2.4. Nutrition and Health Claims of Infant Foods

Table 4 presents information on the nutrition claims in the labelling of infant foods. Results showed that the main claims reported on infant foods labels are related to the presence/absence of added sodium, sugar and fat (according to the national law of the country where the products are marketed) and the claims related to the main fortifying nutrients, i.e., calcium, fiber and proteins. Overall, calcium was found to be the most frequently added nutrient to infant foods, since it is one the crucial nutrients at the infancy stage. Calcium salts (calcium carbonate, calcium phosphates or calcium citrate, Table S1) were used for calcium fortification. Such a fortification is recommended to reach the needs of infants, beside the consumption of other calcium food sources, such as breast milk, vegetables or nuts [31].

Baby formula (0–6 months) have limited number of claims, compared to the rest of the categories, despite it being the category with the highest number of products. Vegetarian baby formula presented claims related to sugar, sodium, fat, sugar, calcium, protein and fiber, whereas vegan baby formula have calcium and protein claims. No claims related to sodium were found in baby formula (6–12 months). Few vegetarian products were sold with claims related to sugar and fat reduction/absence/addition, while none of vegan products made such claims. Notably, 15.38% of this category (vegetarian) presented claims related to calcium fortification, compared to 0.51% of vegan. 

Vegetarian baby biscuits and rusks had higher frequency of products having sodium, sugar and fat-related claims. However, as shown in Table 2, the sugar content in vegetarian products was much higher that vegan. The number of vegetarian products fortified in calcium was also higher than vegan products. Only one product of each category presented a claim related to fiber. Vegan baby cereals had higher frequency of products made with claims related sodium and protein, while vegetarian products were mostly (33.43%) claiming no added/free/low sugar, compared to only 5.23% of those vegans. Similarly, calcium fortification was chiefly claimed in vegetarian products; even though it was based on nutritional facts, no significant difference was found between both group (Table 3). Vegetarian baby snacks presented high frequency of claims related to sugar and sodium, while only few products had such a claim in the vegan category. A total of 5.98% vegetarian products had claims related to calcium fortification, while no vegan product presented this claim, similarly to fiber fortification. As for protein related claims, 3.42% vegetarian products presented this claim.

Vegetarian baby fruit products, desserts and yogurts presented claims in all nutrients with the highest frequency of sugar related claims (11.01%), compared to those vegans having only sugar (0.77%). 

Baby juices and drinks also presented a low number (5 products out of 17) of nutrients-related claims. Vegetarian products presented 5.88% claims related to sugar and 11.76% related to calcium, while 11.76% vegan products had claims related to protein content. 

Vegetarian baby savory meals and dishes presented high frequency of claims related to sodium and sugar (25% and 26.92%, respectively), compared to no product from the vegan category. Both categories had similar frequency of claims related to sodium. Protein-related claims were only found in vegetarian category. 

Table 5 summarizes the top four health claims reported on infant packages. These claims represent infant food’s effect on the structure or function of the body for maintenance of good health and nutrition. Top used claims are related to brain and nervous system, immune system, bone health and digestive health, due to their relevance for a healthy growth. A higher number of vegetarian baby biscuits and rusks had health claims related to brain and nervous system and immune system, compared to vegan products having high products with bone and digestive health claims. Vegetarian baby cereals had a high number (20–37%) of products, with claims related to brain and nervous system, immune system and bone health underlining the relevance of these claims for products marketing. However, vegan products presented lower percentage of products having health claims probably due to the lack of scientific evidence focused on the health benefits of the 100% plant-based diet on children health. Furthermore, the vegan trend is quite recent in the infant industry. Baby formula (0–6 months) did not have numerous products with health claims, compared to baby formula from 6 to 12 months. In particular, for vegetarian baby formula (6–12 months), almost 49% of products had health claims related to brain and nervous and immune systems and around 31% related to bone health. This might be, in part, related to the high number of products claimed with added calcium (around 15%). Vegan products had few products with health claims in this type of product, similarly to what observed in baby fruit products, desserts and yogurts, baby savory meals and dishes, and baby snacks, compared to vegetarian products. For baby juices and drinks, vegan products presented claims related to brain and nervous system and bone health, while none in the vegetarian category had immune system-related claims.

## 3. Materials and Methods

Data collection: The market search of commercial infants’ foods was carried out by consulting the Mintel Global New Product Database (Mintel GNPD-Mintel Group Ltd., London, UK). The Mintel GNPD tracks packaged food and beverage launches in 86 markets worldwide. Each item has detailed product information, such as price, ingredients, claims made and nutritional information, as well as photographs of all sides of the packaging. The Mintel GNPD search was conducted on 1 July 2022.

The search considered the sub-category of “Baby food” out of the super-category of “foods”. The selection criteria were products launched in the last complete 5 years (2017–2021) in the global market. Only infant foods having the nutritional facts related to energy, carbohydrates, sugar, protein, fat, SFA, fiber, sodium, iron and calcium were considered. Based on the list of their ingredients, the collected products were classified into vegan products and vegetarian products (containing animal derivates, Table S1). Nutrition and health claims were also retrieved. The results of all searches were exported to Microsoft Excel (Microsoft Office, Washington, WA, USA). 

Data extraction: Nutritional facts related to energy (kcal/100 g), total fat (g/100 g), saturated fatty acids-SFA (g/100 g), carbohydrates (g/100 g), sugars (g/100 g), protein (g/100 g), fiber (g/100 g), sodium (mg/100 g), calcium (mg/100 g) and iron (mg/100 g) were retrieved, as well as nutrition and health claims. 

Statistical data analysis: The statistical analysis was carried out using the Statistical Package for Social Sciences software (IBM SPSS Statistics, Version 25.0, IBM corp., Chicago, IL, USA). Based on Kolmogorov–Smirnov test, the normality of data distribution was rejected, and, therefore, data were expressed as median values with interquartile ranges 25th–75th percentile. Energy and nutrient contents of products were analyzed using Mann–Whitney non-parametric test (*p*  <  0.05) for two independent samples.

## 4. Conclusions

Vegetarian and vegan infant food from global market were compared in terms of nutritional quality, ingredient formulations, as well as nutritional and health claims. Among the eight identified food categories, baby formula (0–6 months and 6–12 months) covered the largest market share, with vegetarian products that were more often present on the grocery stores shelves. Several plant-based ingredients were used in formulating foods, such as cereals, pulses, seeds and pseudocereals. Compared to their vegetarian counterparts, vegan products (both baby formula and baby juices and drinks) were higher in saturated fat content, whereas vegetarian baby formula (0–12 months) contained less protein amount. Probably due to a fortification strategy, calcium and iron were generally found higher in vegan infant foods. Claims related to brain and nervous system, immune system, as well as bone and digestive health, were more frequently reported on the vegetarian product packaging, as opposed to the vegan ones. Results revealed the current nutritional state of these products are gaining popularity in the segment of infant nutrition. With the aim of ensuring ever better nutritional quality, implementing more research in this field would be of primary importance. These may include the evaluation of the glycemic index and protein quality of the products, as well as clarifying the role of fiber and oligosaccharides within the infants’ organism. Under a technological standpoint, more efforts are needed to understand the role of some ingredients within the complex food matrix of the infants’ food products, in order to put in place successful strategies of ingredient substitution/addition.

## Figures and Tables

**Table 1:** Distribution of infant foods across product categories.

Type	Group	Number of Products	Percentage Out of Total
Infant foods	Vegetarian	2010	91.57%
	Vegan	185	8.43%
	Total	2195	100.00%
Baby formula (0–6 months)	Vegetarian	509	23.19%
	Vegan	61	2.78%
	Total	570	25.97%
Baby formula (6–12 months)	Vegetarian	384	17.49%
	Vegan	6	0.27%
	Total	390	17.77%
Baby biscuits and rusks	Vegetarian	128	5.83%
	Vegan	17	0.77%
	Total	145	6.61%
Baby cereals	Vegetarian	319	14.53%
	Vegan	25	1.14%
	Total	344	15.67%
Baby snacks	Vegetarian	211	9.61%
	Vegan	23	1.05%
	Total	234	10.66%
Baby fruit products, desserts, and yogurts	Vegetarian	347	15.81%
	Vegan	44	2.00%
	Total	391	17.81%
Baby juices and drinks	Vegetarian	13	0.59%
	Vegan	4	0.18%
	Total	17	0.77%
Baby savory meals and dishes	Vegetarian	99	4.51%
	Vegan	5	0.23%
	Total	104	4.74%

**Table 2:** Nutritional facts of commercial infant foods.

Type	Group	Energy (kcal/100 g/mL)	Fat (g/100 g/mL)	Saturated Fat (g/100 g/mL)	Sodium (mg/100 g/mL)
Baby Formula (0–6 months)	Vegetarian	475.14 (66.00–513.00)	20.6 (3.3–27.3)	2.5 (1.1–11.2)	120 (20–210)
	Vegan	498.00 (66.20–516.40)	25 (3.42–28)	10.6 (1.5–13.48)	170 (20–240)
	Significance	***	**	***	**
Baby Formula (6–12 months)	Vegetarian	70.00 (66.05–498.00)	3.5 (3–24.2)	1.4 (0.8–9.4)	30 (20–214.5)
	Vegan	489.50 (65.00–ND)	17.3 (3–ND)	9.85 (1.5–ND)	174 (30–ND)
	Significance	ns	ns	**	*
Baby Biscuits and Rusks	Vegetarian	415 (357.14–450.45)	9.7 (0–15.25)	0 (0–7.5)	174.24 (0–357.14)
	Vegan	414 (400–477.42)	7.2 (0–13.06)	1 (0–4.18)	10 (0–304)
	Significance	ns	ns	ns	***
Baby Cereals	Vegetarian	400 (93.75–437)	7.15 (0–12.9)	1.73 (0–4.5)	80 (0–200)
	Vegan	415 (128.68–425)	9 (0.21–10.6)	4.2 (0–5.08)	115 (3.43–188)
	Significance	ns	ns	*	ns
Baby Snacks	Vegetarian	400.00 (351.42–466.67)	3.9 (0–18.72)	0 (0–5.106)	119.05 (0–355.71)
	Vegan	375.00 (352.8–460.00)	3.55 (0–20)	0 (0–2.25)	0 (0–395.2)
	Significance	ns	ns	ns	*
Baby Fruit Products, Desserts and Yogurts	Vegetarian	66.66 (50.50–98.27)	0 (0–2.94)	0 (0–1.186)	8.84 (0–35.35)
	Vegan	70.70 (50.25–106.19)	0 (0–1.76)	0 (0–0.88)	4.79 (0–23.13)
	Significance	ns	ns	ns	ns
Baby Juices and Drinks	Vegetarian	52.63 (27.03–332.22)	0.52 (0–3.15)	0 (0–0.94)	39.47 (1.00–211.11)
	Vegan	360 (300.00–ND)	1.5 (1–ND)	1 (0.5–ND)	190 (111–ND)
	Significance	**	*	**	ns
Baby Savory Meals and Dishes	Vegetarian	70.80 (35.39–350.00)	1.76 (0–5.05)	0.2 (0–1.35135)	42.96 (5.30–193.54)
	Vegan	70.71 (53.09-ND)	3.03 (0-ND)	0 (0-ND)	30.97 (10.10-ND)
	Significance	ns	ns	ns	ns
Baby Formula (0–6 months)	Vegetarian	50 (7.2–58.1)	0.1 (0–3.6)	7.6 (5.69–55.04)	5.9 (1.28–12.5)
	Vegan	53.02 (7–59)	0.6 (0–4.1)	6.2 (0–24)	11.1 (1.6–13.9)
	Significance	ns	ns	***	***
Baby Formula (6–12 months)	Vegetarian	8.6 (7.45–60.8)	0.1 (0–2.9)	8.2 (5.3–53.5)	1.8 (1.3–11.9)
	Vegan	52.76 (7- ND)	1.5 (0- ND)	3.5 (0- ND)	11.65 (2- ND)
	Significance	ns	*	***	**
Baby Biscuits and Rusks	Vegetarian	75 (66.5–95.24)	0 (0–5.4)	20 (2.86–26.73)	6 (0–10.43)
	Vegan	79.2 (63–82)	2 (0–3.57)	20 (0–29)	7 (3.36–40)
	Significance	ns	ns	ns	*
Baby Cereals	Vegetarian	68.2 (14.84–84)	3 (0–6.67)	25 (4.4–35.7)	12.6 (1.77–15)
	Vegan	68.5 (0.09–82.4)	1.3 (0.15–11.48)	6.67 (0–19.28)	15 (1.89–15.52)
	Significance	ns	ns	***	*
Baby Snacks	Vegetarian	73.33 (57.14–87.5)	0 (0–12.4)	14.28 (0.00–57.14)	7.14 (0–17.85)
	Vegan	60 (53–90)	6.67 (0–12.5)	8.44 (0.00–80.00)	12.5 (0–20)
	Significance	ns	***	ns	ns
Baby Fruit Products, Desserts and Yogurts	Vegetarian	14.43 (11.50–20.58)	1.76 (0.45–3.02)	10.07 (6.79–15.04)	0.90 (0–2.79)
	Vegan	15.54 (11.30–20.35)	1.76 (0.88–4.42)	10.10 (7.84–14.14)	0.95 (0–2.60)
	Significance	ns	ns	ns	ns
Baby Juices and Drinks	Vegetarian	9.47 (6.90–72.22)	1.57 (0–17)	6.31 (4.94–18.33)	3.15 (0–6.55)
	Vegan	68.50 (55-ND)	1 (0.9-ND)	5 (4-ND)	2.5 (1-ND)
	Significance	*	ns	ns	ns
Baby Savory Meals and Dishes	Vegetarian	9.73 (1.69–72.04)	1.76 (0–4)	2.34 (0–6.06)	2.66 (0.88–12)
	Vegan	10.10 (9.40-ND)	2.35 (0.88-ND)	3.03 (1.01-ND)	2.02 (0.00-ND)
	Significance	ns	ns	ns	ns

ND: not determined, * *p*  <  0.05, ** *p*  <  0.01, *** *p*  <  0.001, ns non-significant (*p*  >  0.05).

**Table 3:** Calcium and iron contents of infant foods.

Type	Group	Calcium (mg/100 g/mL)	Iron (mg/100 g/mL)
Baby formula (0–6 months) (*n* = 570)	Vegetarian	300 (44.09–515.5)	1.8 (0.5–6)
	Vegan	426 (51–570)	5.3 (0.67–8.4)
	Significance	***	***
Baby formula (6–12 months) (*n* = 390)	Vegetarian	76.95 (62–560)	1.1 (0.83–7.45)
	Vegan	441.10 (81–nd)	6.44 (1.10–nd)
	Significance	ns	*
Baby biscuits and rusks (*n* = 145)	Vegetarian	28.99 (0.00–545.16)	2.30 (0–11.87)
	Vegan	390.00 (0.00–400.00)	7.00 (0–7.20)
	Significance	*	ns
Baby cereals (*n* = 344)	Vegetarian	433.33 (21.42–620.00)	8.50 (1.30–31.90)
	Vegan	450.00 (66.19–1615.00)	7.90 (0.60–12.10)
	Significance	ns	ns
Baby snacks (*n* = 234)	Vegetarian	105 (0–514.28)	2.5 (0–21.43)
	Vegan	26.26 (0–650)	2.5 (0–4.22)
	Significance	ns	ns
Baby fruit products, desserts and yogurts (*n* = 391)	Vegetarian	14.11 (6.67–67.26)	0.35 (0–1.08)
	Vegan	11.31 (7.80–35.39)	0.73 (0–0.94)
	Significance	ns	*
Baby juices and drinks (*n* = 17)	Vegetarian	86.84 (0–1054.44)	0 (0–21.78)
	Vegan	833.00 (777-nd)	15.5 (12-nd)
	Significance	ns	*
Baby savory meals and dishes (*n* = 104)	Vegetarian	21.21 (0–67.56)	0.70 (0–2.1)
	Vegan	26.26 (12.12–nd)	1.01 (0.60–nd)
	Significance	ns	ns

* p  <  0.05, *** *p*  <  0.001, ns non-significant (*p*  >  0.05), nd: not determined.

**Table 4:** Top nutrition claims of infant foods (expressed in percentage out the total of product type).

Type	Group	Low/No/Reduced Sodium	No Added/Free/low Sugar	Low/No/Reduced Fat	Added Calcium	High/Added Fiber	High/Added Protein
Baby formula (0–6 months) (*n* = 570)	Vegetarian	0.35% (*n* = 2)	0.70% (*n* = 4)	0.23% (*n* = 6)	0.49% (*n* = 13)	0.88% (*n* = 5)	0.88% (*n* = 5)
	Vegan	-	-	-	0.70% (*n* = 4)	-	0.17% (*n* = 1)
Baby formula (6–12 months) (*n* = 390)	Vegetarian	-	3.08% (*n* = 12)	1.28% (*n* = 5)	15.38% (*n* = 60)	1.02% (*n* = 4)	0.51% (*n* = 2)
	Vegan	-	-	-	0.51% (*n* = 2)	-	-
Baby biscuits and rusks (*n* = 145)	Vegetarian	10.34% (*n* = 15)	9.66% (*n* = 14)	1.38% (*n* = 2)	21.38% (*n* = 31)	0.69% (*n* = 1)	0.69% (*n* = 1)
	Vegan	2.76% (*n* = 4)	2.06% (*n* = 3)	0.69% (*n* = 1)	4.82% (*n* = 7)	2.06% (*n* = 3)	0.69% (*n* = 1)
Baby cereals (*n* = 344)	Vegetarian	1.74% (*n* = 6)	33.43% (*n* = 115)	0.87% (*n* = 3)	25.00% (*n* = 86)	-	0.87% (*n* = 3)
	Vegan	3.20% (*n* = 11)	5.23% (*n* = 18)	0.29% (*n* = 1)	2.90% (*n* = 10)	-	1.16% (*n* = 4)
Baby snacks (*n* = 234)	Vegetarian	10.26% (*n* = 24)	12.82% (*n* = 30)	1.28% (*n* = 3)	5.98% (*n* = 14)	1.71% (*n* = 4)	3.42% (*n* = 8)
	Vegan	1.28% (*n* = 3)	0.85% (*n* = 2)	0.43% (*n* = 1)	-	-	0.85% (*n* = 2)
Baby fruit products, desserts and yogurts (*n* = 391)	Vegetarian	5.63% (*n* = 22)	11.01% (*n* = 43)	0.77% (*n* = 3)	2.05% (*n* = 8)	1.02% (*n* = 4)	3.32% (*n* = 13)
	Vegan	-	0.77% (*n* = 3)	-	-	-	0.25% (*n* = 1)
Baby juices and drinks (*n* = 17)	Vegetarian	-	5.88% (*n* = 1)	-	11.76% (*n* = 2)	-	-
	Vegan	-	-	-	-	-	11.76% (*n* = 2)
Baby savory meals and dishes (*n* = 104)	Vegetarian	25.00% (*n* = 26)	26.92% (*n* = 28)	-	2.88% (*n* = 3)	-	4.90% (*n* = 5)
	Vegan	-	-	-	2.88% (*n* = 3)	-	-

*n*= number of products, -: no product was retrieved.

**Table 5:** Top health claims of plant-based infant foods.

Type	Group	Claims Related to Brain and Nervous System	Claims Related to Immune System	Claims Related to Bone Health	Claims Related to Digestive Health
Baby formula (0–6 months) (*n* = 570)	Vegetarian	5.26% (*n* = 30)	5.26% (*n* = 30)	1.75% (*n* = 10)	13.15% (*n* = 75)
	Vegan	1.23% (*n* = 8)	1.22% (*n* = 7)	0.70% (*n* = 4)	1.05% (*n* = 6)
Baby formula (6–12 months) (*n* = 390)	Vegetarian	48.46% (*n* = 189)	48.71% (*n* = 190)	30.77% (*n* = 120)	7.18% (*n* = 28)
	Vegan	1.79% (*n* = 7)	1.53% (*n* = 6)	0.51% (*n* = 2)	1.53% (*n* = 6)
Baby biscuits and rusks (*n* = 145)	Vegetarian	13.10% (*n* = 19)	7.59% (*n* = 11)	3.45% (*n* = 5)	0.69% (*n* = 1)
	Vegan	0.69% (*n* = 1)	6.90% (*n* = 10)	4.83% (*n* = 7)	1.38% (*n* = 2)
Baby cereals (*n* = 344)	Vegetarian	37.80% (*n* = 130)	30.23% (*n* = 104)	20.34% (*n* = 70)	5.81% (*n* = 20)
	Vegan	2.32% (*n* = 8)	1.16% (*n* = 4)	1.16% (*n* = 4)	-
Baby snacks (*n* = 234)	Vegetarian	9.83% (*n* = 23)	2.14% (*n* = 5)	1.28% (*n* = 2)	6.41% (*n* = 15)
	Vegan	2.14% (*n* = 5)	1.28% (*n* = 3)	0.43% (*n* = 1)	-
Baby fruit products, desserts and yogurts (*n* = 391)	Vegetarian	6.14% (*n* = 24)	3.84% (*n* = 15)	3.07% (*n* = 12)	4.09% (*n* = 16)
	Vegan	0.25% (*n* = 1)	0.25% (*n* = 1)	-	0.25% (*n* = 1)
Baby juices and drinks (*n* = 17)	Vegetarian	-	17.65% (*n* = 3)	-	-
	Vegan	23.53% (*n* = 4)	-	23.53% (*n* = 4)	-
Baby savory meals and dishes (*n* = 104)	Vegetarian	11.54% (*n* = 12)	1.92% (*n* = 2)	2.88% (*n* = 3)	-
	Vegan	2.88% (*n* = 3)	-	2.88% (*n* = 3)	-

*n* = number of products, -: no product was retrieved.

## Data Availability

Not applicable.

## Supplementary Materials

The following supporting information can be downloaded at: https://www.mdpi.com/article/10.3390/plants11192531/s1, Table S1: List of the main ingredients of infant foods.
