# Supplementary material for: Nutritional Facts and Health/Nutrition Claims of Commercial Plant-Based Infant Foods: Where Do We Stand?
Source: Plants (Basel). 2022 Sep 27;11(19):2531. doi: 10.3390/plants11192531 (PMC9572996; doi:10.3390/plants11192531)
Supplement: Supplementary file 1 [file plants-11-02531-s001.zip › plants-1885010-supplementary.pdf]

Table S1. List of the main ingredients of infant foods

Sub-Category	Vegetarian	Vegan
Baby Formula (0-6 months)		
Carbohydrates and sugar	Maltodextrin; Dried Glucose Syrup;	
Vegetable Oils	Corn Oil; Rapeseed Oil; Palm Oil; Soybean Oil; Coconut Oil; Sunflower Seed Oil - High Oleic Acid	
Plant proteins	Isolated Soy Protein;	
Minerals	Manganese Sulphate; Copper Sulfate; Zinc Sulfate; Calcium Carbonate; Calcium Chloride; Magnesium Phosphates; Phosphorus; Magnesium; L-tyrosine; Potassium Hydrogen Carbonate; Calcium Pantothenate; Calcium; Zinc; Tripotassium Phosphate; Copper; Selenium; Sodium; Choline; Iron; Calcium Citrates; Sodium Citrate; Potassium Hydroxide; Magnesium Chloride; Potassium Chloride; Ferrous Sulphate; Calcium Phosphates; Manganese; Potassium; Chloride; Iodine;	
Vitamins	Vitamin B6; Vitamin A; Vitamin D; Vitamin B12; Niacin; Vitamin B1; Vitamin C; Vitamin K; Carnitine; Riboflavin; Folic Acid; Biotin; Vitamins; Tocopherol; Vitamin E;	
Dairy	Semi-skimmed Milk; Whey Powder; Skim Milk; Whey Protein Concentrate; Whey Protein; Full Fat Milk; Lactose;	-
Baby Formula (6-12 months)		
Carbohydrates and sugar	Glucose; Dried Glucose Syrup; Maltodextrin; Dextrin	
Vegetable Oils	Coconut Oil; Corn Oil; Sunflower Seed Oil; Soybean Oil; Rapeseed Oil;	
Dairy	Lactose; Skimmed Milk Powder; Whey Protein Concentrate; Full Fat Milk; Semi-skimmed Milk; Whey Powder;	-
Vitamins	Vitamin A; Vitamin B12; Riboflavin; Vitamin E; Vitamin B6; Vitamin B1; Vitamin K; Vitamin C; Beta-Carotene;	
Minerals	Calcium Citrates; Cytidine 5'-monophosphoric Acid; Disodium 5'-uridine; Calcium Pantothenate; Disodium Inosinate; Copper Sulfate; Potassium Hydroxide; Magnesium Chloride; Zinc Sulfate; Sodium Citrate (Food Potassium; Potassium Chloride; Manganese Sulphate; Calcium Carbonate; Manganese; Chloride; Potassium Iodide; Ferric Ammonium Citrate; Zinc; Phosphorus; Magnesium; Tripotassium Phosphate; Copper;	
Plant proteins	Rice Protein; Isolated Soy Protein;	
Baby Biscuits & Rusks		
Carbohydrates and sugar	White Sugar; Glycerol; Glucose<br>Inulin;	
Cereals and legumes	Wheat Flour; Barley Malt Extract; Red Rice; Brown Rice; Rice Flour; Flour; Oats; Barley Flour; Pea Protein; Rye Flour; Quinoa; Oat Flour; Finger Millet; Rice; Rye; Corn; Chickpea Flour; Wheat Flavour; Cornflours; Wheat Starch; Potato Starch; Barley;	
Vegetables and fruits	Blueberry Pulp; Cherry; Fruit Pulp; Sweet Potato; Broccoli; Apple Puree; Banana Puree; Banana; Fig Pulp; Beetroot;	
Vitamins	Vitamin A; Riboflavin; Vitamin D; Vitamin B1; Vitamin E; Vitamin B6; Vitamin K; Vitamin B12;	
Minerals	Iron; Zinc Sulfate; Ammonium Carbonates; Ferrous Fumarate; Sodium Phosphates;	
Vegetable Oils	Palm Oil; Margarine	
Seeds	Linseed; Chia Seed	
Dairy	Butter; Yogurt; Milk Powder; Cheese and Cheese Products; Skimmed Milk Powder; Milk Proteins;	-
Nuts	Almond	

Baby Cereals		
Carbohydrates and sugars	White sugar; starches and dextrin; glucose syrup; sweeteners;	
Cereals	Rice flour; barley malt, oat flour; wheat flour; corn flours; rice; sugars and other carbohydrate; rolled oats; whole wheat flour; millet flour; green gram flour;	
Dairy	Milk; milk solids; whole milk powder; milk proteins; butter ghee; skimmed milk powder;	-
Vegetable and fruits	Orange Juice Concentrate: Blackcurrant Juice Concentrate; Banana; Apple Juice Concentrate; Apple; Pomegranate Juice Concentrate; Mango Powder; Mango Pulp; Carrot; Spinach ; Lemon Powder ; Broccoli ; Cherry Puree; Blueberry; Tomato Puree; Raisins	
Vegetable oils	Sunflower Seed Oil	
Minerals	Iron; Zinc Sulfate Copper; Copper Sulfate; Calcium; Zinc; phosphorus; Iodine; Magnesium; Taurine; Selenium;	
Vitamins	Vitamin C; Vitamin A; Vitamin E; Vitamin B6; Vitamin B1; Vitamin D; Vitamin B12	
Baby Snacks		
Carbohydrates and sugar	Cane Sugar; Brown Sugar; Cassava Starch; Maltodextrin; White Sugar Pea fiber, inulin	
Cereals and legumes	Wheat; Wheat Starch; Jasmine Rice; Riceberry Rice; Oats; Cornflours; Barley Malt Extract; Rice; Rice Flour; Sorghum Flour; Whole Wheat Flour; White Rice Flour; Quinoa Flour; Lentil Flour; Pea Flour; Chickpea Flour; Corn; Cassava Flour; Green Lentil; Yellow Pea;	
Vegetable oils	Sunflower Seed Oil; Rice Bran Oil; Safflower Seed Oil; Palm Oil;	
Vegetables and fruits	Carrot Powder; Spinach; Carrot; Blueberry Powder; Chinese Broccoli; Banana Powder; Apple Powder; Apple; Beetroot; Strawberry; Banana; Onion; Broccoli Powder; Carrot Extract; Parsley; Broccoli; Pomegranate; Cranberry Powder; Apple Extract; Raspberry; Pumpkin Powder; Raisins; Spinach Powder; Blueberry; Berries and Other Small Fruits; Pomegranate Powder; Raspberry Powder;	
Dairy	Cheddar Cheese; Milk; Butter Milk (Butter; Skimmed Milk Powder; Processed Cheddar Cheese; Food) Skim Milk;	-
Vitamin	Vitamin E	
Minerals	Calcium	
Baby Fruit Products, Desserts & Yogurts		
Pulses and cereals	Chickpea; Pea Protein Isolate; Wheat; Oat Flour; Peas; Pea Protein; Kidney Bean; Black Bean; Oat Milk; Quinoa Flour; Blackberry; Mango Puree; Carob Bean Gum; Quinoa Milk;	
Seeds	Linseed; Chia Seed;	
Fruits and vegetables	Pumpkin; Orange Juice Concentrate; Kale; Kiwifruit; Apricot Puree; Grapes; Zucchini; Beetroot; Carrot Puree; Coconut Cream; Berries and Other Small Fruits; Raspberry; Passion Fruit Juice; Raisins; Fig; Pineapple; Citrus Pectin; Concord Grapes; Pineapple Juice Concentrate; Peach Puree; Cranberry Puree; Purple Potato; Strawberry Puree; Pear Puree; Carrot; Blueberry Puree; Banana; Apple Puree; Strawberry (Food Date; Coconut Milk; Lemon Juice Concentrate; Coconut; Lemon Juice; Apple; Banana Puree; Navy Bean; Butternut Squash; Vitamin C; Sweet Potato; Mango; Pear; Apricot; Spinach; Purple Carrot Extract; Pumpkin Puree; Blueberry;	
Dairy	Greek Yogurt	-
Baby Juices & Drinks		
Carbohydrates and sugars	White Sugar, Maltodextrin, Caramel	
Cereals	Wheat flour, wheat Gluten, Barley, Millet	
Dairy	Milk Solids	-

Minerals	Potassium Hydrogen Carbonate	
Plant-based proteins	Isolated Soy Protein	
Baby Savory Meals & Dishes		
Carbohydrates and sugar	Maltodextrin<br>Inulin	
Cereals and legumes	Rice Flour; Peas; Rice; Millet; Green Lentil; Black Bean; Cassava Flour; Brown Rice Flour; Corn flours; Durum Wheat Semolina; Corn; Sunflower Seed Oil; White Bean; Quinoa; Sweet Corn;	
Fruits and vegetables	Cauliflower; Apple; Carrot; Onion; Basil; Spinach; Carrot Puree; Pumpkin; Apple Puree; Tomato Paste; Broccoli; Sweet Potato; Lemon Juice Concentrate; Tomato;	
Vitamin	Folic Acid; Niacin; Vitamin D; Vitamin A; Vitamin B12; Riboflavin; Vitamin C; Vitamin E; Vitamin B6; Vitamin B1;	
Minerals	Calcium Carbonate; Zinc Sulfate; Iron;	
Dairy	Milk Proteins; Cheese and Cheese Products; Skimmed Milk Powder; Milk; Whole Milk Powder;	-
Vegetable oils	Palm Oil; Extra Virgin Olive Oil	
